# Validity evaluation of the genetics and genomics in nursing practice survey

**DOI:** 10.1002/nop2.346

**Published:** 2019-08-13

**Authors:** Alexandra Plavskin, William E. Samuels, Kathleen A. Calzone

**Affiliations:** ^1^ Hunter College New York New York; ^2^ College of Staten Island CUNY Staten Island New York; ^3^ Center for Cancer Research National Institutes of Health, National Cancer Institute Bethesda Maryland

**Keywords:** genetics, genomics, instrument development, statistical modelling

## Abstract

**Aim:**

To psychometrically test the Genetics and Genomics Nursing Practice Survey (GGNPS) for evidence of content, face and construct validity.

**Design:**

This study was a secondary data analysis.

**Method:**

Data collected from the Method for Introducing a New Competency into Nursing Practice (MINC) study were used to evaluate the GGNPS for evidence of construct validity via structural equation modelling and confirmatory factor analysis. Face validity was evaluated via feedback from practicing RNs without specific experience with or knowledge of genetics/genomics. Content validity was evaluated via content expert feedback and assessment of a content validity index.

**Results:**

The thresholds for evidence of content and face validity were met. However, we found less evidence for construct validity of the GGNPS; an exploratory factor analysis, conducted to gain additional insight into the theorized latent constructs, determined that the variables were more interrelated that previously predicted.

## 1 INTRODUCTION

Genetics and genomics play key roles in nursing practice. These roles include providing patient and family education, administering prescribed treatments, advocating disease prevention/health promotion, collecting and interpreting family history information, and collaborating with the healthcare team to facilitate genetic referrals if warranted. These roles and responsibilities demonstrate that Registered Nurses (RNs) in all specialties, clinical roles and practice settings must adopt genetics/genomics into their practice (Jenkins & Calzone, 2007; Starkweather et al., 2018).

Due to the importance of genetics/genomics in clinical practice, the Genetics and Genomics Nursing Practice Survey (GGNPS) was created to evaluate RNs’ competency/knowledge, attitudes/receptivity, confidence, and decision/adoption of genetics and genomics into nursing practice; in addition, the GGNPS evaluates the effect of social systems in the healthcare setting on all of these domains. The instrument was developed from a well‐validated instrument assessing the adoption of genetics/genomics by family physicians (FPs) (Jenkins, Woolford, Stevens, Kahn, & McBride, 2010). Revising the original FP instrument to reflect nursing practice created the GGNPS. While the FP instrument was evaluated using content expert feedback and structural equation modelling, the GGNPS has not had same validity evaluation.

## 2 BACKGROUND

Genetics and genomics have a significant impact on the leading causes of mortality and morbidity and must be a component of evidence‐based, competent nursing practice aiming to improve patient outcomes (National Academies of Sciences, Engineering, and Medicine, 2016). Genetics/genomics affect aspects of care in all clinical roles and nursing specialties, making it a required competency of nursing practice (Calzone, Jenkins, Culp, Caskey, & Badzek, 2014; Consensus Panel on Genetic/Genomic Nursing Competencies, 2009). The GGNPS can play an important role as researchers assess nurses’ knowledge and use of genetics/genomics in clinical practice.

A number of instruments relating to genetics/genomics are available, but focus predominantly on education, perceived (as opposed to objective) knowledge or do not focus specifically on nursing practice. For example, the Genetics and Genomics in Nursing State of the Science Conference Survey focuses on nurse educators and faculty. This instrument contains questions about genetics/genomics courses taught in nursing curricula, whether participants’ certification examinations contained questions related to genetics/genomics, continuing education and current research (Thompson & Brooks, 2011).

The Genetics Needs Assessment Survey (Maradiegue, Edwards, Seibert, Macri, & Sitzer, 2005) is a 70‐item instrument evaluating participants’ perceived knowledge of and comfort with genetics as well as desired educational activities to increase knowledge (e.g. lectures, role play, problem sets). It does not assess objective genetics knowledge via questions that can be marked as correct/incorrect; it also does not assess associated concepts such as attitudes towards genetics/genomics, or use in clinical practice.

Additional instruments, such as the Genomic Nursing Concept Inventory (GNCI), measure understanding of genetic/genomic topics (Ward, 2011). The GNCI was developed by surveying baccalaureate nursing students. It focuses on evaluation of knowledge and has been predominantly used with students and faculty. Bottorff et al. (2005) developed an instrument to evaluate knowledge, confidence and professional roles in providing genetic services for adult‐onset hereditary diseases. The instrument was created and tested on nurses and physicians in Canada. Lastly, researchers in the United Kingdom created an instrument evaluating nurses’ confidence and knowledge about collecting family history information, as well as providing referrals (Bankhead et al., 2001). While a variety of genetics/genomics‐based instruments are available, the GGNPS focuses on nursing practice. It also evaluates how factors such as confidence, social systems and attitudes interact with knowledge to ultimately lead to adoption or rejection of genetics/genomics in clinical practice.

The GGNPS is internationally relevant because the adoption benchmark is family history (FH) collection. Collecting FH information is not dependent on access to technology or funding. This benchmark was specifically selected by the developers of the GGNPS to allow it to be used in a variety of countries and areas—especially regions with variable resources (Calzone et al., 2012). Additionally, the Global Genomics Nursing Alliance (G2NA) conducted an international survey, including representatives from 18 countries, and it included FH taking as a key component of nursing roles in the delivery of genomic services (Calzone, Jenkins, Jenkins, Culp, & Badzek, 2018; Calzone, Kirk, et al., 2018). The aim of this study was to evaluate the GGNPS for evidence of content, face and construct validity.

## 3 DESIGN

This is a secondary analysis of baseline data previously collected during the MINC study.

### 3.1 Survey instrument

The GGNPS was initially pilot tested for usability with a convenience sample (5 nurses), then tested again with a larger sample (Calzone et al., 2012). A second pilot test was conducted with 239 RNs employed by the National Institutes of Health (NIH). Following pilot testing, the GGNPS was revised based on participant feedback and a review by content experts; these revisions included the removal of questions that expert reviewers considered unclear. It also included the addition of two items about knowledge of genetics of common diseases (added with permission) from the Genetic Variation Knowledge Assessment Index (Bonham, Sellers, & Woolford, 2014).

In addition, the GGNPS underwent reliability testing using data from two clinical institutions (Calzone et al., 2016). The reliability testing study included baseline and completion assessments from a 1‐year educational intervention. Participants completed the GGNPS twice, at the beginning and at the end of the intervention. The study found that Likert‐scale items with five or more options had poor test–retest reliability; scales of those items were shortened to increase reliability.

Calzone et al. (2016) reported that the mean agreement across all items in the GGNPS was “moderately” strong (mean Cohen's *κ* ≈ .41), as defined by Landis and Koch (1977, p. 165). Of the test–retest κs, 39% (*n* = 36/95) were between .41 and .60. However, some of the items had substantial variability in reliability, ranging from .150 to 1. Calzone et al. (2016) also found that “Select‐all‐that‐apply” items related to self‐assessment of one's ability to discuss genetics of common diseases with clients performed the poorest. Items pertaining to attitude/receptivity also performed nearly as poorly. In addition, they found that Likert‐scaled items with fewer response options, 3–4 options, performed better than Likert‐scaled items with 7 response options, regardless of item content.

The version of the GGNPS used for this study did not include revisions made following reliability testing because reliability testing was completed after baseline data were collected in the Method for Introducing a New Competency into Nursing Practice (MINC) study (Calzone et al., 2016). Following an educational intervention, reliability testing was conducted to obtain a sample in which participants would answer questions based on understanding of genetics/genomics. If the baseline data were used for reliability testing, reliability could also be affected by participants’ lack of knowledge about genetics/genomics. In simplest terms, reliability may be affected if participants were guessing while completing the GGNPS. The current study complements Calzone and colleagues’ investigations into the reliability of the instrument with research into several aspects of its validity.

### 3.2 Sample/Participants

The sample included 7,798 RNs from 23 Magnet^®^ hospitals in 17 states from all regions of the United States. Magnet^®^ hospitals were selected because they are believed to provide higher quality of nursing care; Kalisch and Xie (2014) reported increased patient teaching, additional staffing resources and improved communication in Magnet^®^ hospitals compared to comparable non‐Magnet^®^ hospitals. Due to these characteristics, Magnet^®^ hospitals have an increased capacity to support both innovation and pilot programs for new initiatives (Calzone et al., 2014); therefore Magnet^®^ hospitals were selected as part of purposive sampling.

The final sample, following analysis for missing data, consisted of staff nurses (53%, *n* = 3,638/6,861) whose primary role was direct patient care (61%, *n* = 4,186/6,861); 42% (*n* = 2,888/6,861) reported spending 81%–100% of their time seeing patients (Table 1). This sample is representative of the target population because the GGNPS is intended to evaluate the use of genetics/genomics by nurses providing direct patient care (Tables 1 and 2).

**Table 1:** Frequency table—clinical experience

Variable	Frequency	Percent of participants (%)
Primary area of expertise		
Case manager	96	1.40
Clinical nurse specialist	110	1.60
Consultant	47	0.69
Director/Assistant director	109	1.59
Educator	230	3.35
Head nurse	286	4.17
Nurse practitioner	185	2.70
Researcher	41	0.60
Staff nurse	3,638	53.02
Supervisor	232	3.38
No response	1,887	27.50
Percent time seeing patients		
0%–20%	741	10.8
21%–40%	256	3.73
41%–60%	374	5.45
61%–80%	741	10.8
81%–100%	2,888	42.1
No response	1,861	27.12
Number of years working in nursing		
0–3 years	1,137	16.57
6–10 years	744	10.84
11–15 years	606	8.83
16–20 years	643	9.37
21–25 years	502	7.32
26–30 years	587	8.56
31–50 years	954	13.91
No response	1,688	24.60

**Table 2:** Frequency table—demographic variables

Variable	Frequency	Percent of participants (%)
Gender		
Female	4,875	71.05
Male	328	4.78
No response	1,658	24.17
Ethnicity/Race		
American Indian/Alaska Native	26	0.38
Asian	384	5.60
Black/African American	335	4.88
Native Hawaiian/Pacific Islander	34	0.50
White	4,272	62.26
No response	1,810	26.38
Hispanic/Latino		
Yes	231	3.37
No	4,950	72.15
No response	1,680	24.49
Age		
20–45 years	2,354	34.31
46–65 years	2,328	33.93
66+ years	92	1.34
No response	2,087	30.42
Highest Nursing Degree		
Diploma	324	4.72
Associate Degree in Nursing	1,062	15.48
Baccalaureate Degree in Nursing	3,065	44.67
Master's Degree in Nursing	731	10.65
Doctorate Degree in Nursing	33	0.48
No response	1,646	23.99

## 4 METHOD

### 4.1 Conceptual framework

The conceptual framework was based on Rogers’ (2003) diffusion of innovations (DOI) model. Rogers describes diffusion of innovations as a process or series of processes in which an individual, or group, initially acquires knowledge about an innovation, forms a favourable or unfavourable attitude about it and then selects to either adopt or reject it.

The study variables were derived from Rogers’ model and work by Calzone et al., 2014, 2012. The “competency/knowledge” variable addressed gaining an understanding of genetics/genomics and being able to use it in the scope of RNs’ professional standards and responsibilities. The “attitude/receptivity” variable evaluated nurses’ beliefs and perceptions about genetics/genomics. “Social systems” was the variable that explored the setting and environment where this innovation was being used. Lastly, “decision/adoption” addressed the decision to adopt or reject genetics/genomics and how this innovation was used in nursing practice. Each variable was operationally and conceptually defined by mapping the item to the associated variable for statistical modelling (Table 3).

**Table 3:** Conceptual and operational definition of variables

GGNPS variable	Conceptual definition	Operational definition
Competency/Knowledge	Competency: Individuals providing safe patient care, in accordance with “responsibilities, professional standards, education, and qualifications” (Axley, 2008, p. 221). Knowledge: “when an individual (or other decision‐making unit) is exposed to an innovation's existence and gains some understanding of how it functions.”	Part 2, Questions 2.1, 2.2, 2.4; Part 4, Questions 1, 2, 3; Part 5, Questions 1, 2; Part 6, Questions 1, 2.
Attitude/Receptivity	Attitude: “A relatively enduring organization of an individual's beliefs about an object that predisposes his or her actions” (Rogers, 2003, p. 174–175). Receptivity: “favorable or unfavorable attitude towards an innovation” (Rogers, 2003, p. 169). While knowledge is mostly 'knowing' or cognitive, attitude/receptivity is mostly 'feeling' (Rogers, 2003).	Part 1, Questions, 1, 2, 3; Part 2, Questions 2, 3.
Decision/Adoption*	Decision: activities that lead to a choice of either adopting or rejecting the innovation. Adoption: use of an innovation (Rogers, 2003). In the present study, decision/adoption was operationally defined as self‐reported collection and assessment of a family history, as well as self‐reported facilitation of referrals to genetic services.	Part 3, Questions 2, 3, 4.
Confidence	“Level of certainty that knowledge about the innovation is accurate” (Calzone et al., 2012, p. 12).	Part 2, Question 1.
Social system	Social system: the setting or environment where the innovation was introduced such as the clinical site where nurses are employed (Calzone et al., 2012).	Part 7, Questions 1.3, 1.4, 1.5, 1.6, and 1.7.

* Decision was the portion of the DOI model where individuals engaged in activities that affected the choice of adopting or rejecting an innovation. However, the decision stage is difficult to observe and measure because decisions are often internal thoughts (Rogers, 2003). For this reason, Calzone and colleagues combined decision and adoption into one domain, facilitating empirical measurement. In this study, decision was considered part of decision/adoption and is not measured separately.

Confidence was included as a variable based Calzone et al., 2014, 2012, Calzone, Jenkins, Culp, Bonham, and Badzek (2013), although it is not part of the DOI model. It was included based on Rogers’ (2003) description of how personality traits can affect adoption of an innovation. Rogers reported that earlier adopters have a more positive attitude towards change and are better able to cope with uncertainty and risk. Furthermore, Benner’s (1984) description of confidence in nursing paralleled many characteristics reported by Rogers. Benner reported that experienced nurses are more confident in their abilities and are more capable of navigating difficulties/challenges. As nurses come closer to becoming experts, they increase their critical thinking ability; critical thinkers exhibit a number of characteristics including confidence, flexibility and open‐mindedness (Benner, Hughes, & Sutphen, 2008). This parallels Rogers’s (2003) theory individuals who are more capable of coping with uncertainty and risk are more likely to adopt innovations. As a result, this study evaluated the direct effect of competency/knowledge on confidence, the direct effect of confidence on attitudes/receptivity, as well as the indirect effect of confidence on decision/adoption.

### 4.2 Outcome indicators

GGNPS indicators of adoption of genetics/genomics included both collection and evaluation of a family history, facilitating referrals to specialists as needed and applying knowledge of clinical genetics/genomics to provide competent and current patient care. These indicators of adoption are applicable to nurses of all levels of academic preparation in a variety of clinical areas, in varying specialties, and with variable access to technology. Collection and evaluation of a family history, patient education and facilitation of referrals are applicable for nurses caring for clients in both in‐patient and community settings. They are also applicable for RNs working with patients across the lifespan, using paper or electronic documentation systems, and with varying access to sequencing and related genetic technology (Calzone et al., 2012).

The variables in this study were defined based on work by Calzone et al., 2014, 2012 and associated research. The defined variables were used to create a proposed model, based on both Rogers’ DOI model and on research related to adoption of genetics/genomics to nursing practice.

### 4.3 Hypothesized model

**Figure 1:**
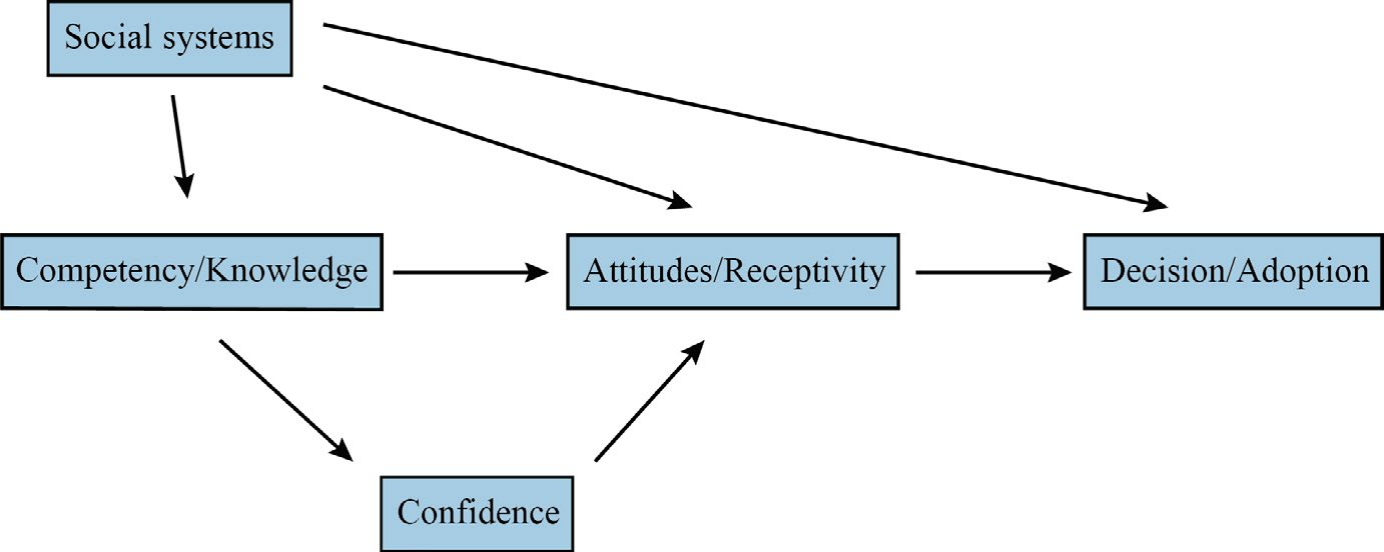
Hypothesized model

The hypothesized model was created based on the GGNPS, Rogers’ (2003) DOI model and literature related to adoption of genetics/genomics into nursing practice (see Figure 1). Rogers described both the importance and pervasive nature of social systems; as a result, we hypothesized that social systems would have both direct and indirect relationships with all other variables in the model. We additionally hypothesized that competency/knowledge would directly affect the formation of attitudes/receptivity of the innovation. This could, in turn, affect adoption of the innovation. Lastly, we hypothesized that one's self‐reported level of competency/knowledge would affect one's level of confidence, which could also influence attitudes/receptivity.

### 4.4 Ethical considerations

Institutional review board (IRB) approval for this study was obtained from the primary investigator's institution, protocol number 2016‐0067. The IRB allowed an exemption from full review because data were not collected with identifying information, ensuring confidentiality.

### 4.5 Data analysis

Data were analysed using Mplus version 7.3 (Muthén & Muthén, 2012) and R version 3.1.2 (R Core Team, 2013). R packages used included nortest (Gross & Ligges, 2015), psych (Revelle, 2018), MVN (Korkmaz, Goksuluk, & Zararsiz, 2014) and mice (van Buuren & Groothuis‐Oudshoorn, 2011), along with code written by the investigators to let R interface with Mplus.

### 4.6 Missing data

Of 7,798 participants, 8.71% (*n* = 679) respondents left all items in the instrument blank and were excluded. This left 7,119 respondents. Data were then inspected for respondents who answered predominantly demographic items, but omitted a majority of the other questions; calculating a ratio of demographic items answered to total items answered. Individuals who had a *z*‐score of ± 3 or greater were excluded (*n* = 3). These individuals answered predominantly demographic questions, without providing information pertinent to evaluation of the study variables.

The remaining 7,116 responses were reviewed for participants who answered the majority of items pertaining to one variable (e.g. confidence), but not to the other variables. Ratios were calculated between complete data on individual variables versus the entire instrument; individuals who had a *z*‐score of ±3 or greater were excluded (*n* = 262); 7 individuals had a z‐score of ±3 in relation to both confidence and decision/adoption as compared to the entire instrument. Following removal of those responses, the final sample included 6,861 participants.

Lastly, data from the remaining 6,861 respondents were examined for patterns of missingness. Welch's two‐sample independent *t* tests were used to determine whether data could be considered missing at random (MAR): that is, whether there was no statistically significant mean difference in one variable (e.g. social systems) if an item in a different variable (e.g. confidence) was unanswered. Before conducting *t* tests, marginal mean imputations were conducted; without imputation, the *t* tests would provide information about the *frequency* of missing data, as opposed to relations between missing items. Šidák family‐wise error correction was used to determine the family‐wise α‐values for multiple *t* tests. Missingness on decision/adoption items could not be considered to be MAR, while missingness on other variables did appear to be MAR.

Multiple imputation, the most statistically robust method (Carpenter, Kenward, & Vansteelandt, 2006), was used to account for missing items because not all data were MAR. Imputations were conducted using the mice (van Buuren & Groothuis‐Oudshoorn, 2011) package in R, because the GGNPS contains a variety of items including dichotomous ‘yes/no’ items, Likert‐scaled items and an interval‐level knowledge score. By beginning every imputation with the variable that has the least amount of missing data, the mice package utilizes the most complete variables to fill in data on less complete variables. Lastly, the mice package also uses random draws of data to mimic natural variability in the data.

Four imputations, with 50 iterations for each imputation, were conducted. The multiple iterations allow the program to try multiple variations until a “best‐fit” scenario is selected. ANOVAs compared means of the four imputations. Results demonstrated that there were no statistically significant differences between the imputations, demonstrating increased likelihood that the data were imputed based on the parameters of the existing data.

## 5 RESULTS

### 5.1 Content validity

Evidence of content validity was investigated using a content validity ratio (CVR). Eight experts in both genetics/genomics and nursing were provided a content validity index (CVI) that contained the items in the GGNPS. The experts were individuals who have expertise, current employment and evidence of scholarship in both genetics/genomics and nursing. Experts with backgrounds in clinical practice, education and/or genetics/genomics research (as opposed to solely bench science) were selected, because the GGNPS aims to evaluate clinical nursing practice. The CVI included each item in the GGNPS along with the definition of each domain. Each item was scored using a relevance table with four categories (ranging from “very relevant” to “not relevant”) (Lawshe, 1975). The CVR was calculated using Lawshe's formula:

$$CVR=[N_{e}-\left(N/2\right)]/\left(N/2\right)$$

where *N_e_* is the number of panellists who classified the item as “very relevant” or “relevant” and *N* is the total number of content experts. The threshold for supporting content validity for the GGNPS was met. All content experts completed and returned the CVI. One responder answered the items intended for the learners, not the content experts; feedback from this responder was excluded. The remaining seven responses were used to calculate the CVR.

A higher CVR indicates greater evidence of content validity. A value of 1 indicates that all reviewers considered an item “very relevant” or “relevant”. A comprehensive CVI was calculated by taking the mean of content validity ratios across all items. CVR values for individual items ranged from −0.714 to 1. The overall CVI was 0.805, exceeding 0.741, the value Wilson, Pan, and Schumsky (2012) reported as the required threshold when seven content experts provide feedback.

Wilson et al. (2012) derived their calculations in attempting to recreate Lawshe (1975) and Schipper's seminal work using discrete binomials and normal approximation to the binomial. While Lawshe reported critical CVR at *α* = 0.05, one tailed, values calculated by Wilson and colleagues were closer to normal approximation to the binomial at *α* = 0.05, two tailed (or *α* = 0.025, one tailed). As a result, when calculated by Wilson et al. (2012), the critical CVR for *n* = 7 was 0.741 (*p* = .05, two tailed test). A CVR of 0.741 (*n* = 7) is achieved if only one content expert rates an item as “somewhat relevant” or “not relevant”. Therefore, both calculating the CVR using normal approximation to the binomial and consideration that all, except one, content experts consider the item “very relevant” or “relevant” are the basis for establishing support for content validity.

### 5.2 Face validity

Seven reviewers, all nurses with a variety of backgrounds, provided feedback for face validity evaluation (Table 4). The reviewers were selected to provide the perspective of the majority of nurses in the workforce; therefore, none had specific education or expertise in genetics/genomics. Because the GGNPS was created for use in clinical nursing practice, reviewers whose primary role was patient care were selected. Face validity evaluated the ease of understanding and applying the instrument to nursing practice (Table 4). The threshold for acceptable support for face‐related validity for the GGNPS was met.

**Table 4:** Face validity results

Number of years working in nursing	Proportion time seeing patients (%)	Primary area of practice	Highest nursing degree	Is the instrument clear and easy to understand?	Are the questions pertinent to your clinical practice?
30	100	Staff nurse	AS	Very clear/easy	No
4	100	Staff nurse (ICU)	BS	Somewhat clear/easy	Yes
2	60	Staff nurse (ER)	BS	Somewhat clear/easy	No
30	100	Staff nurse	DNP	Somewhat clear/easy	Yes
31	40	Educator	MS	Very clear/easy	Yes
21	100	Staff nurse	MS	Somewhat clear/easy	Yes
10	70	Head nurse (Med/Surg)	MS	Somewhat clear/easy	Yes

### 5.3 Construct validity

Approximately 12% of the data in the original data set were missing; although this amount of missingness is common, we used multiple imputation to ensure the most accurate and robust results possible. We then tested the model structure on each of the four imputed sets and the original data. Conducting the CFA with five datasets allowed comparison between versions of imputed data as well as robust tests of the original model.

The CFA loadings are not reported because the CFA failed to converge on stable model parameters. Examining the results found that model parameters appeared not to converge largely because of items’ varied response structures; variance accounted for in the model itself was eclipsed by variance due to item structure (e.g. dichotomous yes/no items, multiple choice). As a result, analyses could not converge on sufficiently stable model parameters. Some items contained little variance while others contained much more. Additionally, relationships between items appear more complicated than the factor model could explain.

### 5.4 Structural equation model

The CFAs, therefore, could neither adequately support nor refute the theoretical model. To further investigate construct‐related validity of the GGNPS, we conducted a series of structural equation models (SEMs) evaluating the relation between factors and the associated evidence for construct‐related validity.

Using the model, *χ* ^2^ from the *SEM* analyses as an initial fit test (Table 5) found that the model did not fit the data well (*χ* ^2^ = 451.979, *p* < .001). However, *χ* ^2^ statistics are sensitive to sample size (Hooper, Coughlan, & Mullen, 2008) and provide one, limited perspective on the SEMs. The root mean square error of approximation (RMSEA) was used to evaluate model fit, while also considering sample size. The RMSEAs for the SEMs used the original data set, and the four variations of imputed data suggest the models still did not provide what Browne and Cudeck (1993) would consider a good fit (Table 5).

**Table 5:** Model fit using the original data set and multiple imputations

	Original data set	Imputed 1	Imputed 2	Imputed 3	Imputed 4
Chi‐square	451.979 *df* = 3 *p* < .001	603.970 *df* = 3 *p* < .001	657.126 *df* = 3 *p* < .001	617.126 *df* = 3 *p* < .001	593.837 *df* = 3 *p* < .001
RMSEA	0.170	0.160	0.167	0.162	0.159
CFI	0.839	0.875	0.871	0.872	0.877
BIC	79,506.863	139,302.730	139,205.026	139,625.503	139,297.560

The comparative fit index (CFI) was used to gain further insight into the SEMs by comparing how well the model structure proposed in the *SEM* compares against a null model. An incremental fit index, a CFI ≥0.9 indicates a good model fit (Davis & Murrell, 1993). The CFI was not >0.9 in the original data or the four variations of imputed data (Table 5).

Lastly, we computed Bayesian information criteria (BICs) for each *SEM*. BIC corrects for both the sample size and number of parameters in a model and (Raftery, 1993). BIC is a comparative fit measure between models, with a lower BICs indicating better model fit. Among the original data set and the four variations of imputed data, the BICs ranged from 79,507 to 139,625 (Table 5).

**Figure 2:**
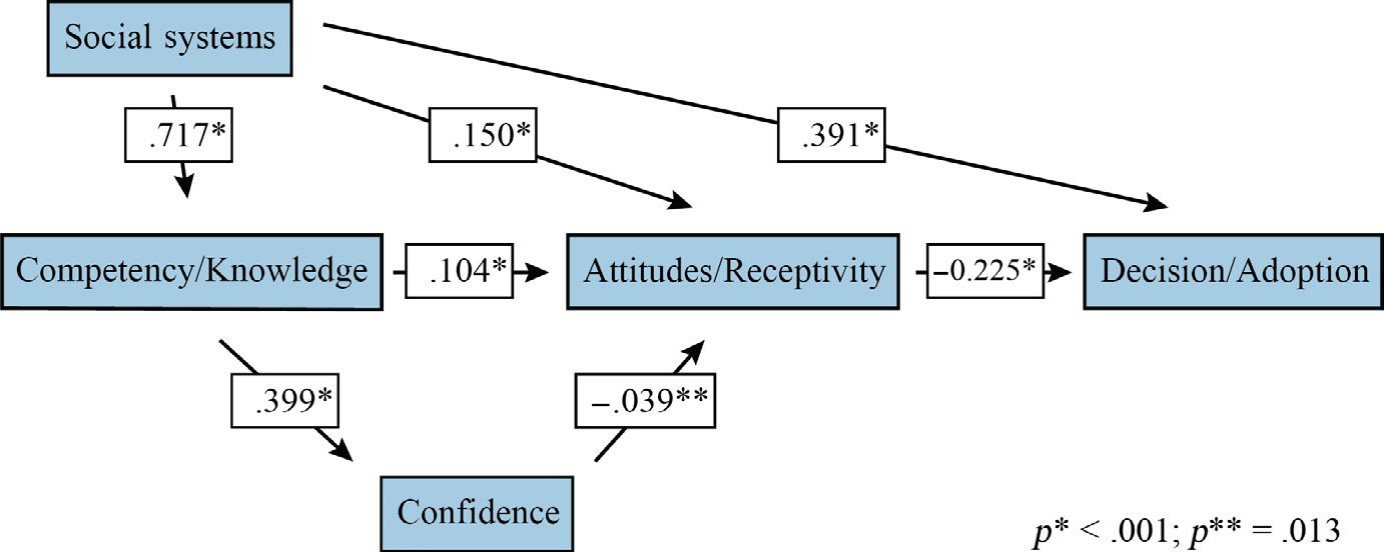
Structural equation model

Although reported, the relationships among the variables in *SEM* must be interpreted cautiously because numerous fit analyses all indicated that the hypothesized model did not fit the data well (see Figure 2). Although there was a statistically significant direct effect of competency/knowledge on attitudes/receptivity (0.104), this relation was weak when the large sample size was considered. Meanwhile, the direct effect of competency/knowledge on confidence was one of the strongest relationships in the *SEM* (0.399). The relation between attitudes/receptivity on decision/adoption was weak and negative (−0.225). The relation between social systems and competency/knowledge (0.717) was the strongest in the *SEM*. By contrast, the relation between attitudes/receptivity and social systems (0.150) was weak, but it was statistically significant. The direct relation between attitudes/receptivity and confidence (−0.039) was the weakest relation in the *SEM*. Lastly, the direct relation between social systems and decision/adoption was one of the strongest in the *SEM* (0.391). The large sample size increases the likelihood that statistically significant correlations exist among the data. When considered together, the large sample size, the weak relationships and the results of the fit indices, all illustrate that the hypothesized model did not fit the data well (Wolf, Harrington, Clark, & Miller, 2013).

### 5.5 Ancillary analysis

Thresholds for evidence of content and face validity were met; however, we did not find strong evidence for construct validity. Since we did not find sufficient evidence that the items on the GGPNS produce a theoretically sound model, we conducted an exploratory factor analysis (EFA) to investigate the factor structure. We used five factors to reflect the number used in the *SEM*; we also used oblique factor rotation to allow factors to be related to each other (Osborne, 2015). Criteria used for evaluation were factor loadings ≥0.30 (Costello & Osborne, 2005) and presence of Thurstone's (1947) simple structure. The EFA results demonstrate that the factors were more interrelated than anticipated; these results can be used to re‐evaluate which items are related to respective DOI factors and used to inform future construct validity analyses.

## 6 DISCUSSION

Content and face validity for the GGNPS were well supported. However, the threshold for construct validity was not met. The inability of the CFA to produce factor loading is related to the varied structure and relationships between the items. The response structure of the GGNPS, specifically the variety of questions (e.g. categorical and dichotomous items), created fluctuating amounts of variance among the items that affected the ability to evaluate the variance of the overall model. For example, items with more answer choices contained higher variance than dichotomous items.

Additionally, the relationships between the variables may be more complex than predicted in the hypothesized model. This complexity appears to prevent us from establishing construct validity: the GGNPS is a complex instrument attempting to measure multiple domains in different ways. The complexity was a barrier in attempting to establish construct validity. Additionally, because the EFA showed that many items designated in the GGNPS as one variable actually loaded on a different variable (Table 6), this can create a measurement error that would be compounded because the *SEM* evaluated direct and indirect relations amongst five variables (Table 7). Even if a small number of items in each variable actually measured a different variable, this would create non‐negligible measurement error.

**Table 6:** Model fit using the original data set and multiple imputations

GGNPS designated factor	EFA designated factor	EFA overview
Confidence	Factor 1: Confidence	Confidence items consistently load onto Factor 1
Attitudes/Receptivity	Factor 2: Attitudes/Receptivity	Attitudes/receptivity items and items from competency/knowledge that are related to nurses’ attitudes regarding genetics/genomics load onto Factor 2
Decision/Adoption	Factor 3: Decision adoption	Decision/adoption items and items from attitudes/receptivity that are related to perceived disadvantages of adopting genetics/genomics into nursing practice load onto Factor 3
Competency/Knowledge	Factor 4: Competency/Knowledge	Many competency/knowledge items load onto Factor 4, specifically items related to collection of family history information
Competency/Knowledge	Factor 5: Competency/Knowledge	Competency/knowledge items also load onto Factor 5, specifically items related to clinical decision‐making
Social systems	Did not load as a variable	Social system items did not consistently load onto any single factor, some items loaded on two other factors, while other items did not load above .30.

**Table 7:** Model fit using the original data set and multiple imputations indirect path model relations

	Original data	Imputation 1	Imputation 2	Imputation 3	Imputation 4
Competency/Knowledge & Decision/Adoption	−0.023	−0.020	−0.030	−0.023	−0.026
Social systems & attitudes/Receptivity	0.075	0.039	0.061	0.046	0.050
Social systems & decision/Adoption	−0.034	−0.084	−0.091	−0.083	−0.082
Confidence & Decision/Adoption	0.0088	0.0084	0.011	0.015	0.012

The previously conducted evaluation of reliability also provides insight into the weak relations in the *SEM*. Many attitude/receptivity items, especially “Select‐all‐that‐apply” items or those that included large Likert scales, were the most poorly performing items during reliability testing (Calzone et al., 2016). The low reliability of these items led researchers to revise or eliminate a number of those questions. Notably, variables endogenous and exogenous to the attitudes/receptivity variable were the weakest relationships in the *SEM*, for example, the relationship between competency/knowledge and attitudes receptivity (0.104); attitudes/receptivity and decision/adoption (−0.225); confidence and attitudes/receptivity (−0.039); and social systems and attitude receptivity (0.150). Relations that included attitudes/receptivity as either the dependent or the independent variable were all weaker than other relations in the hypothesized model.

Evaluation of construct validity also demonstrated key parallels between the GGNPS and the FP instrument. In the *SEM* analysis of the original instrument, used by FPs, Jenkins et al. (2010) also reported that adoption of genetics/genomics did not match the path in the DOI model. Researchers reported two paths leading to adoption of genomic‐related innovations: one path based on “comfort” with using genetics/genomics in clinical practice and a second path based on “relevance” to clinical practice. The researchers hypothesized that the multiple paths may be related to the complexity of clinical practice and the complexity of the innovation, genetics/genomics. This complexity was a contrast to the linear nature of Rogers’ DOI model. These varied paths to adoption of genetics/genomics can provide insight into further evaluations of construct validity in the GGNPS. Together with the EFA results, this will be used to redefine the GGNPS variables and evaluate paths to adoption of genetics/genomics in nursing practice.

### 6.1 Limitations

Although a common limitation among research based in healthcare settings, survey burden and time constraints, resulting in data missingness, were notable limitations in this study. RNs may initially express interest in participating, but realize they do not have adequate time to complete the GGNPS or consider all of the items carefully. In fact, 679/7,798 participants (8.71%) opened the instrument to complete it, but left all items blank. Furthermore, all of the institutions used for data collection had conducted institution‐wide nursing data collection in the previous 6 months (Calzone et al., 2014). Survey burden may affect not only the accuracy of responses, but may also contribute to measurement error. Multiple imputation, a statistically rigorous method to address missing data, was used to decrease the limitations caused by partially completed surveys.

## 7 CONCLUSION

Analyses support GGNPS face and content validity. However, construct validity remains to be supported. The CFA and *SEM* models proposed in this study did not fit the data well. EFA results in the ancillary analysis provide both insight into the likely sources of measurement error and recommendations for revision and retesting of the GGNPS.

## CONFLICT OF INTEREST

There are no conflicts of interest to disclose.

## References

[nop2346-bib-0001] Axley, L. (2008). Competency: A concept analysis. Nursing Forum, 43(4), 214–222. 10.1111/j.1744-6198.2008.00115.x 19076465

[nop2346-bib-0002] Bankhead, C. , Emery, J. , Qureshi, N. , Campbell, H. , Austoker, J. , & Watson, E. (2001). New developments in genetics—knowledge, attitudes and information needs of practice nurses. Family Practice, 18(5), 475–486. 10.1093/fampra/18.5.475 11604367

[nop2346-bib-0003] Benner, P. (1984). From novice to expert. The American Journal of Nursing, 82(3), 402–407. 10.1097/00000446-198412000-00027 6917683

[nop2346-bib-0004] Benner, P. , Hughes, R. G. , & Sutphen, M. (2008). Clinical reasoning, decision‐making, and action: Thinking critically and clinically In Hughes R. G. (Eds.), Patient safety and quality: An evidence‐based handbook for nurses. Rockville, MD: Agency for Healthcare Research and Quality (US). 21328752

[nop2346-bib-0005] Bonham, V. L. , Sellers, S. L. , & Woolford, S. (2014). Physicians’ knowledge, beliefs, and use of race and human genetic variation: New measures and insights. BMC Health Services Research, 14(1), 456 10.1186/1472-6963-14-456 25277068 PMC4283084

[nop2346-bib-0006] Bottorff, J. L. , Blaine, S. , Carroll, J. C. , Esplen, M. J. , Evans, J. , Nicolson Klimek, M. L. , … Ritvo, P. (2005). The educational needs and professional roles of Canadian physicians and nurses regarding genetic testing and adult onset hereditary disease. Community Genetics, 8(2), 80–87. 10.1159/000084775 15925883

[nop2346-bib-0007] Browne, M. W. , & Cudeck, R. (1993). Alternative ways of assessing model fit. Sage Focus Editions, 154, 136–136.

[nop2346-bib-0008] Calzone, K. A. , Culp, S. , Jenkins, J. , Caskey, S. , Edwards, P. B. , Fuchs, M. A. , … Badzek, L. (2016). Test‐retest reliability of the genetics and genomics in nursing practice survey instrument. Journal of Nursing Measurement, 24(1), 54–68. 10.1891/1061-3749.24.1.54 27103245 PMC4883680

[nop2346-bib-0009] Calzone, K. A. , Jenkins, J. , Culp, S. , & Badzek, L. (2018). Hospital nursing leadership‐led interventions increased genomic awareness and educational intent in Magnet settings. Nursing Outlook, 66(3), 244–253. 10.1016/j.outlook.2017.10.010 29544651 PMC5949252

[nop2346-bib-0010] Calzone, K. A. , Jenkins, J. , Culp, S. , Bonham, V. L. , & Badzek, L. (2013). National nursing workforce survey of nursing attitudes, knowledge and practice in genomics. Personalized Medicine, 10(7), 719–728. 10.2217/pme.13.64 PMC3866033 24363765

[nop2346-bib-0011] Calzone, K. A. , Jenkins, J. , Culp, S. , Caskey, S. , & Badzek, L. (2014). Introducing a new competency into nursing practice. Journal of Nursing Regulation, 5(1), 40–47. 10.1016/S2155-8256(15)30098-3 25343056 PMC4204730

[nop2346-bib-0012] Calzone, K. A. , Jenkins, J. , Yates, J. , Cusack, G. , Wallen, G. R. , Liewehr, D. J. , … McBride, C. (2012). Survey of nursing integration of genomics into nursing practice. Journal of Nursing Scholarship, 44(4), 428–436. 10.1111/j.1547-5069.2012.01475.x 23205780 PMC3515630

[nop2346-bib-0013] Calzone, K. A. , Kirk, M. , Tonkin, E. , Badzek, L. , Benjamin, C. , & Middleton, A. (2018). The global landscape of nursing and genomics. Journal of Nursing Scholarship, 50(3), 249–256. 10.1111/jnu.12380 29608246 PMC5959047

[nop2346-bib-0014] Carpenter, J. R. , Kenward, M. G. , & Vansteelandt, S. (2006). A comparison of multiple imputation and doubly robust estimation for analyses with missing data. Journal of the Royal Statistical Society: Series A (Statistics in Society), 169(3), 571–584. 10.1111/j.1467-985X.2006.00407.x

[nop2346-bib-0015] Consensus Panel on Genetic/Genomic Nursing Competencies (2009). Essentials of genetic and genomic nursing: Competencies, curricula guidelines, and outcome indicators (2nd ed.). Silver Spring, MD: American Nurses Association.

[nop2346-bib-0016] Costello, A. B. , & Osborne, J. W. (2005). Best practices in exploratory factor analysis: Four recommendations for getting the most from your analysis. Practical Assessment, Research & Evaluation, 10(7), 1–9.

[nop2346-bib-0017] Davis, T. M. , & Murrell, P. H. (1993). A structural model of perceived academic, personal, and vocational gains related to college student responsibility. Research in Higher Education, 34(3), 267–289. 10.1007/BF00991846

[nop2346-bib-0018] Gross, J. , & Ligges, U. (2015). notest: Tests for normality. Retrieved from https://CRAN.R-project.org/package=nortest.

[nop2346-bib-0019] Hooper, D. , Coughlan, J. , & Mullen, M. R. (2008). Structural equation modeling: Guidelines for determining model fit. Electronic Journal of Business Research Methods, 6(1), 53–60.

[nop2346-bib-0020] Jenkins, J. , & Calzone, K. A. (2007). Establishing the essential nursing competencies for genetics and genomics. Journal of Nursing Scholarship, 39(1), 10–16. 10.1111/j.1547-5069.2007.00137.x 17393960 PMC10461169

[nop2346-bib-0021] Jenkins, J. , Woolford, S. , Stevens, N. , Kahn, N. , & McBride, C. M. (2010). Family physicians’ likely adoption of genomic‐related innovations. Case Studies in Business, Industry and Government Statistics, 3(2), 70–78.

[nop2346-bib-0022] Kalisch, B. J. , & Xie, B. (2014). Errors of omission: Missed nursing care. Western Journal of Nursing Research, 36(7), 875–890. 10.1177/0193945914531859 24782432

[nop2346-bib-0023] Korkmaz, S. , Goksuluk, D. , & Zararsiz, G. (2014). MVN: An R package for assessing multivariate normality. The R Journal, 6(2), 151–162.

[nop2346-bib-0024] Landis, J. , & Koch, G. (1977). The measurement of observer agreement for categorical data. Biometrics, 33, 159–174. 10.2307/2529310 843571

[nop2346-bib-0025] Lawshe, C. H. (1975). A quantitative approach to content validity. Personnel Psychology, 28(4), 563–575. 10.1111/j.1744-6570.1975.tb01393.x

[nop2346-bib-0026] Maradiegue, A. , Edwards, Q. T. , Seibert, D. , Macri, C. , & Sitzer, L. (2005). Knowledge, perceptions, and attitudes of advanced practice nursing students regarding medical genetics. Journal of the American Academy of Nurse Practitioners, 17(11), 472–479. 16248880 10.1111/j.1745-7599.2005.00076.x

[nop2346-bib-0027] Muthén, L. K. , & Muthén, B. O. (2012). Mplus user’s guide (Version 7th ed.). Los Angeles, CA: Muthén & Muthén.

[nop2346-bib-0028] National Academies of Sciences, Engineering, and Medicine . (2016). Applying an implementation science approach to genomic medicine: Workshop summary. Washington, DC: The National Academies Press. 27123510

[nop2346-bib-0029] Osborne, J. W. (2015). What is rotating in exploratory factor analysis. Practical Assessment, Research & Evaluation, 20(2), 1–7.

[nop2346-bib-0030] R Core Team (2013). R: A language and environment for statistical computing (Version 2.3). Vienna, Austria: R Foundation for Statistical Computing.

[nop2346-bib-0031] Raftery, A. E. (1993). Bayesian model selection in structural equation models. Sage Focus Editions, 154, 163–163.

[nop2346-bib-0032] Revelle, W. (2018) psych: Procedures for personality and psychological research. Evanston, IL: Northwestern University Retrieved from https://CRAN.R-project.org/package=psychVersion=1.8.4

[nop2346-bib-0033] Rogers, E. M. (2003). Diffusion of Innovations (5th ed.). New York, NY: Free Press.

[nop2346-bib-0034] Starkweather, A. R. , Coleman, B. , de Mendoza, V. B. , Hickey, K. T. , Menzies, V. , Fu, M. R. , … Harper, E. (2018). Strengthen federal and local policies to advance precision health implementation and nurses’ impact on healthcare quality and safety. Nursing Outlook, 66(4), 401–406. 30031545 10.1016/j.outlook.2018.06.001

[nop2346-bib-0035] Thompson, H. J. , & Brooks, M. V. (2011). Genetics and genomics in nursing: Evaluating essentials implementation. Nurse Education Today, 31(6), 623–627. 10.1016/j.nedt.2010.10.023 21093123 PMC3117062

[nop2346-bib-0036] van Buuren, S. , & Groothuis‐Oudshoorn, K. (2011). mice: Multivariate imputation by chained equations in R. Journal of Statistical Software, 45(3), 1–67. Retrieved from https://www.jstatsoft.org/v45/i03/.

[nop2346-bib-0037] Ward, L. D. (2011). Development of the genomic nursing concept inventory.

[nop2346-bib-0038] Wilson, F. R. , Pan, W. , & Schumsky, D. A. (2012). Recalculation of the critical values for Lawshe’s content validity ratio. Measurement and Evaluation in Counseling and Development, 45(3), 197–210. 10.1177/0748175612440286

[nop2346-bib-0039] Wolf, E. J. , Harrington, K. M. , Clark, S. L. , & Miller, M. W. (2013). Sample size requirements for structural equation models: An evaluation of power, bias, and solution propriety. Educational and Psychological Measurement, 73(6), 913–934. 10.1177/0013164413495237 PMC4334479 25705052

